# The Dentists

**Published:** 1850-11

**Authors:** 


### ARTICLE III.

### THE DENTISTS.

It is gratifying to see with what zeal the cultivators of the kindred profession of dentistry are exerting themselves to induce those who practice their art to qualify themselves for a scientific and honorable standing.

Our country can boast of two well-organized Colleges of Dental Surgery, the older one being located in Baltimore and the other at Cincinnati. They give thorough instruction in all that pertains to the art, and upon a compliance with their requirements confer the degree of D. D. S., i. e., Doctor of Dental Surgery, on their students. This should be a certificate of qualification, and we should always prefer the dentist who had, to one who had not the degree, unless we had satisfactory means of judging between them independent of this.

We were induced to make these remarks by receiving several documents from the schools above referred to. The addresses of Doctors Hullihen and Townsend to the graduating class of the Baltimore College of Dental Surgery are well drawn up and replete with good sense and good advice.

